# Mycoplasma infections in children: an update

**DOI:** 10.1186/1824-7288-40-S1-A76

**Published:** 2014-08-11

**Authors:** Anna R Cappiello, Maria F Mastrototaro, Mariacristina Pignatelli, Gianluca Piccolo, Iolanda Chinellato, Fabio Cardinale

**Affiliations:** 1 Scuola di Specializzazione in Pediatria, Università degli Studi di Bari “A. Moro”, Bari, 70121, Italy; 2 Università degli Studi di Bari “A. Moro”, Bari, 70121, Italy; 3 Unità Operativa di Pediatria e Fibrosi Cistica, Ospedale “Tatarella”, Cerignola (Foggia), 71042, Italy; 4 Unità Operativa Complessa di Pediatria, Servizio di Pneumologia e Immuno-allergologia Pediatrica, Azienda Ospedaliero-Universitaria “Policlinico-Giovanni XXIII”, Bari, 70126, Italy

## 

Mycoplasma pneumoniae (MP) is a human bacterium that lacks a cell wall and is adapted to life as an obligate pathogen in the respiratory tract [1]. MP is responsible of 20-40% of all community acquired pneumonia (CAP) and is often associated with other airway disorders. Extrapulmonary manifestations are supposed to be sequelae of primary MP infections [2]. MP is believed to first act as an extracellular parasite. However the microbial factors responsible for the observed host cell injury have not been satisfactorily determined [3]. In 2006, a 68-kDa protein CARDS-Tx has been identified [2] from Kannan and others, with a strong cytolytic and vacuolizating activity. Techasaernsiri et al. demonstrates that CARDS-Tx concentrations in BAL of mice inoculated intranasally with three different MP strains, was directly linked to the ability of specific MP strains to colonize, replicate, persist and elicit lung histopathology damage [3]. Furthermore Medina et al. found that CARDS-Tx can induce increased expression of IL4, IL13 and Th-2 chemokines, causing cellular inflammatory response, mucus metaplasia and increase in airway hyperreactivity [4].

On the basis of this observation some authors suggest the use of systemic steroids in order to diminish the host response in MP infection. Observational data, also, indicate that the addition of systemic steroids to antibiotics may improve the outcome of severe MP pneumonia.

Tagliabue et al. demonstrates, in mice, that combination therapy with clarithromycin and dexamethasone is more effective in reducing MP induced pulmonary inflammation than either clarithromycin alone or dexamethasone alone. The authors, however, conclude that more controlled clinical studies in humans are necessary [5].

Macrolide (ML) are recognized as first-choice agents for MP infections. In 2000, however, MP showing resistance to macrolides was isolated from clinical samples obtained from Japanese children with CAP. Since then, prevalence of ML resistant MP isolates in pediatric patients has increased rapidly worldwide.

ML inhibit MP protein synthesis by binding to domain V of 23S rRNA at nucleotide positions 2063 and 2064. Mutations at A2063 or A2064 confer the highest resistance to these antimicrobials [6].

On the basis of studies of the prevalence of ML resistance Principi et al suggest that in countries with low incidence of ML-resistant MP strains no change in ML prescription is initially needed. Nevertheless, in countries in which ML-resistant MP strains are very common, replacement of a ML with a tetracyclines or fluoroquinolones should be considered also based on the severity of the disease [7].
